# Pain at intrauterine device placement in a Brazilian center

**DOI:** 10.1002/ijgo.71026

**Published:** 2026-04-21

**Authors:** Ana Luiza Savi, Judith Lieber, Cássia Raquel Teatin Juliato, Rachel Scott, Luis Bahamondes

**Affiliations:** ^1^ Faculty of Epidemiology and Population Health London School of Hygiene and Tropical Medicine London UK; ^2^ Clinical Trials Unit, Department of Medical Statistics London School of Hygiene and Tropical Medicine London UK; ^3^ Department of Obstetrics and Gynecology Universidade Estadual de Campinas Faculty of Medical Sciences Campinas SP Brazil

**Keywords:** Brazil, contraception, intrauterine devices, long‐acting reversible contraception, pain

## Abstract

**Objective:**

This study explores pain at intrauterine device (IUD) placement in a large Brazilian cohort.

**Methods:**

We undertook a cross‐sectional analysis of health records from individuals aged 18 to 45 years who underwent IUD placements between 2022 and 2024 at the Department of Obstetrics and Gynecology, Universidade Estadual de Campinas Faculty of Medical Sciences, Campinas, Brazil. Insertion pain was measured immediately after the procedure using a 10‐cm visual analogue scale (VAS). Scores were classified as no pain (0), mild pain (1–3), moderate pain (4–6), and severe pain (≥7). Pain relief included analgesics, antispasmodics, non‐steroidal anti‐inflammatory drugs (NSAIDs), and opioids. We used multivariable regression to assess the association between pre‐insertion pain relief and pain during placement.

**Results:**

We evaluated 6974 individuals. Of 7259 fittings analyzed, 228 (3%) occurred with no pain, 1121 (15%) with mild pain, 2003 (28%) with moderate pain, and 3907 (54%) with severe pain (median [interquartile range] pain score of 7.0 [5.0–8.0]). Clinicians provided pain relief in 429 (6%) of insertions (largely antispasmodics and NSAIDs). After adjusting for age and parity, antispasmodics were associated with moderate to severe pain (adjusted odds ratio [aOR] 1.75; 95% confidence interval: 1.15; 2.68).

**Conclusion:**

Our findings suggest that moderate to severe pain was experienced in 5910 (81%) of IUD insertions, in contrast with the 5% estimate in national guidelines. We also found evidence that pre‐insertion antispasmodic medication was associated with higher moderate to severe pain. Our findings emphasize the need to update guidelines to recognize IUD insertion pain and provide options to manage discomfort.

## 1 INTRODUCTION

Intrauterine devices (IUDs) are highly safe and effective contraceptives, with higher contraceptive continuation than short‐term methods.^1^ Despite the availability of copper (Cu)‐IUDs in the public sector, IUDs accounted for only 4.4% of all Brazilian contraceptive choices in 2019.^2^

Many individuals experience pain during IUD placement, and concerns about discomfort remain a barrier to IUD uptake.^3^, ^4^, ^5^ Additionally, users frequently feel inadequately prepared for potential discomfort, receive no information on pain management options, and find the procedure more painful than expected.^6^, ^7^ These experiences are shaped by provider care, as clinicians often underestimate insertion pain, potentially affecting both counseling and pain relief provision.^7^, ^8^

Pain is individual, multifactorial, and might be difficult to predict.^5^ Yet, Brazilian guidelines describe the procedure as uncomplicated and state that “although many women experience some discomfort, less than 5% experience moderate or acute levels of pain.”^9^ Conversely, local studies have estimated that moderate to severe pain occurs in 44% to 81% of IUD insertions.^3^, ^4^, ^10^, ^11^

Despite growing awareness of placement pain, discomfort dismissal, and lack of analgesic options, there is no standard of care to manage insertion discomfort.^5^, ^6^, ^12^ In 2025, the American College of Obstetricians and Gynecologists issued guidance urging clinicians to consider pain‐mitigation strategies during in‐office gynecological procedures, including IUD insertions, and to involve users in decision‐making about pain management.^13^ Additionally, the World Health Organization (WHO) has updated its contraceptive recommendations to include the provision of routine medication to ease IUD placements.^1^ Nevertheless, Brazilian guidelines note that no interventions have been proven to reduce insertion pain.^9^

To help guide clinical decision‐making and promote user‐centered contraceptive care, our study seeks to assess the impact of IUD placement and pain relief on pain experiences in a large Brazilian cohort.

## 2 MATERIALS AND METHODS

We performed a retrospective cross‐sectional analysis of pain experienced during intrauterine device (IUD) insertion at the Department of Obstetrics and Gynecology, Universidade Estadual de Campinas Faculty of Medical Sciences, Campinas, Brazil, from 2022 to 2024. Our analysis used a large sample of routinely collected health records. The study was approved by the Research Ethics Committee of UNICAMP (CAAE no. 7.678.319) and by the London School of Hygiene and Tropical Medicine (LSHTM) MSc Research Ethics Committee (32117). The clinic is a public facility that offers free contraceptive care, as a part of the National Health System. At this facility, providers use standardized forms to collect routine data on IUD insertions, which are stored in a local database.

The Brazilian research team shared anonymized datasets containing IUD insertions between January 2020 and December 2024 under a waiver of informed consent. We restricted the data to individuals aged 18–45 years because of ethical considerations regarding parental consent and few placements in individuals aged over 45 years in the clinic. We excluded observations whose reason for insertion included pelvic pain to minimize confounding by pre‐existing pelvic pain. We also excluded observations from those who underwent the procedure under regional or general anesthesia or sedation, those with missing pain scores, insertions of the 19.5‐mg levonorgestrel IUD (Kyleena, Bayer Oy, Turku, Finland), and unsuccessful insertions. Although we conducted an analysis at the insertion level, 292 (3.2%) observations were missing values for unique individual identifiers, and we estimate that 6974 individuals were included.

All individuals who had an IUD insertion and passed the inclusion criteria were included. Pain scores were recorded alongside other clinical and provider variables, including age, parity, mode of delivery, insertion reasons, type of IUD, type of pre‐insertion pain relief medication, type of insertion provider, insertion difficulty, and cervical dilator use.

Trained providers performed all IUD placements according to the criteria established by WHO^14^ and preferably within the first 5 days of the menstrual cycle to reduce the chance of pregnancy. A pregnancy test was required for individuals with amenorrhea. User‐reported pain scores were measured immediately after insertion using a 10‐cm visual analogue scale (VAS), where 0 was equivalent to “no pain” and 10 to “worst pain ever.” In accordance with previous studies, we categorized pain scores as follows: <1, no pain; 1–3, mild pain; 4–6, moderate pain; and ≥7, severe pain.^4^, ^8^, ^15^ Additionally, we created a binary variable for moderate and severe pain, grouping scores <4 as “no moderate to severe pain” and ≥4 as “moderate to severe pain.”

Age groups (“18–24,” “25–29,” “30–34,” “35–39,” and “40–45”), parity (“0,” “1,” “2,” and “3+”), any history of cesarean deliveries, any vaginal births, and reasons for insertion (“contraception,” “abnormal uterine bleeding,” “endometriosis,” “myoma,” and “other”) were recorded. Care‐related factors included provider (“medical doctor,” “OBGYN resident,” “student,” and “nurse”), type of IUD (“hormonal” or “copper”), pre‐insertion medication (“pain relief” and “no pain relief”), pain relief type (“analgesics,” “antispasmodics,” “NSAIDs” [non‐steroidal anti‐inflammatory drugs] and “opioids”), insertion difficulty (“no difficulty” and “any difficulty”), and use of cervical dilator.

Three types of IUD are offered by the clinic, but only two types were included in the analyses due to infrequent use of the Kyleena (LNG‐IUS 19.5 mg; Bayer Oy, Turku, Finland) device. The hormonal intrauterine device (LNG‐IUS 52 mg; Mirena, Bayer Oy, Turku, Finland) measured 32 × 32 mm and was supplied through a donation program, as this specific model is not commercially available. For this model, insertion is performed using a two‐handed technique, with an inserter tube diameter of 4.8 mm, which is larger than the one‐handed inserter used in the commercially available private‐sector model (4.4 mm). The Cu‐IUD (TCu30A; Optima, Injeflex, São Paulo, Brazil) measured 32 × 36 mm, with an inserter tube diameter of 4.4 mm. The Cu‐bearing IUD reached a 6.2‐mm diameter above the tube when prepared for insertion.^4^ Cervical dilation was performed using an Os finder with gradual dilation from 3 to 5 mm in diameter approximately.

There is no established protocol for pre‐insertion pain relief at the clinic, and pain management options were offered at the provider's discretion between 60 and 15 min before the procedure. We analyzed four types of oral pain relief medications, including analgesics, antispasmodics, NSAIDs, and opioids. No local anesthetics were employed. More details on medications can be found in Table S3.

We used descriptive statistics to explore the distribution of pain outcomes (median and interquartile range for the continuous scores and percentages for the categorical outcomes). To assess whether pain groups differed by characteristic, we employed *χ* ^2^‐tests and reported the corresponding *P*‐values. We also assessed the association between type of pain relief medication and moderate to severe pain using multivariable logistic regression models with a significance level of ≤5%.

We selected confounders based on factors that have previously been associated with insertion pain but are not in the causal pathway, as illustrated in the directed acyclic graph (Figure S1). Each potential confounder was included individually, and those that yielded notable changes in odds ratio (OR) were included in the adjusted model. We employed Stata 19 (College Station, TX: StataCorp LLC) software for statistical analyses.

## 3 RESULTS

All insertions performed between January 2020 and December 2024 were initially considered (*n* = 9279). However, due to the substantial reduction in clinical activity during the COVID‐19 pandemic, few procedures were performed between January 2020 and December 2021. Therefore, we restricted the final analysis to insertions carried out between January 2022 and December 2024. As illustrated in Figure 1, our study assessed 7259 individual IUD insertions.

**FIGURE 1:**
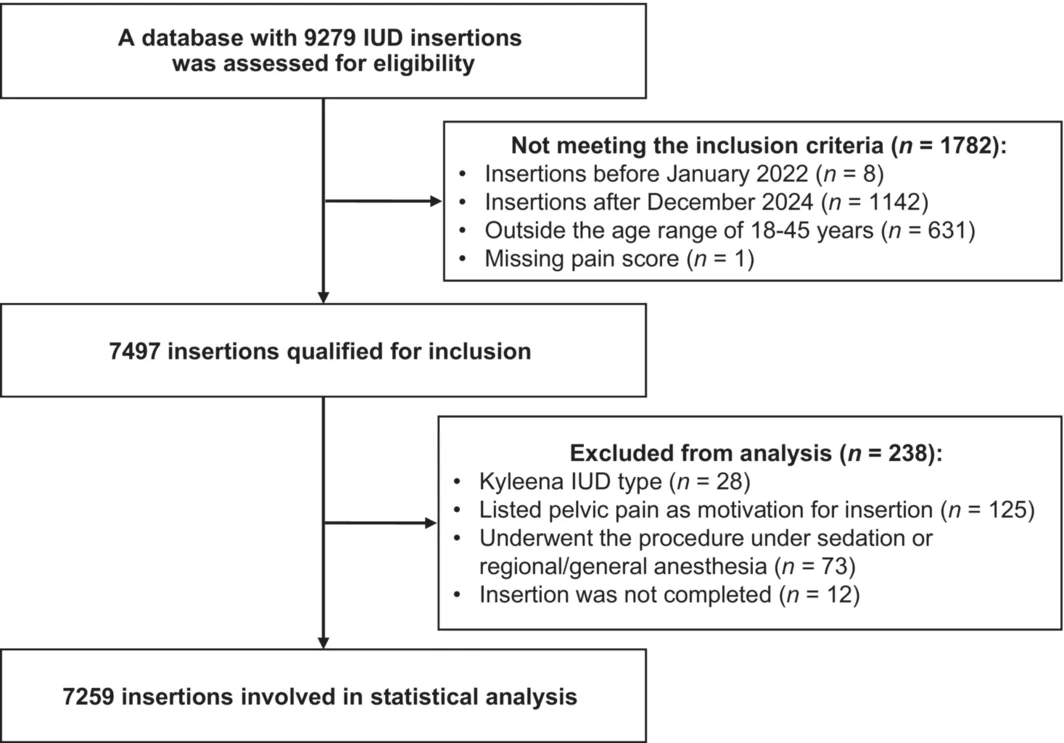
Eligibility flow diagram.

Table 1 summarizes pain outcomes during IUD insertion. The median pain score was 7.0 (interquartile range 5.0–8.0), and most participants experienced moderate to severe pain. Severe pain was reported by 53.8% of individuals, whereas 27.6% reported moderate pain. Mild pain and no pain were uncommon, occurring in 15.4% and 3.1% of cases, respectively.

**TABLE 1:** Pain scores during IUD insertion (*n* = 7259), Campinas, Brazil.

Pain outcome	All
	*n* (%)
Pain scores (0–10)	
Median (IQR)	7.0 (5.0–8.0)
Pain groups	
No pain (0)	228 (3.1)
Mild pain (1–3)	1121 (15.4)
Moderate pain (4–6)	2003 (27.6)
Severe pain (≥7)	3907 (53.8)

Abbreviation: IUD, intrauterine device.

Individual and service‐level characteristics are shown in Table 2. Among participants, 3886 (53.9%) were aged between 18 and 29 years, 4572 (63.4%) were parous, 3957 (54.8%) had experienced at least one vaginal birth, and 2731 (37.8%) had a history of cesarean deliveries. Most insertions were done for contraception (6287, 86.6%). Hormonal IUDs represented 87.5% of all insertions, which were mainly performed by OBGYN residents (2975, 41.1%) and considered “not difficult” (6693, 93%). Clinicians provided pre‐insertion pain relief in 429 (6.1%) cases and used a cervical dilator in less than 1% of fittings. Among the insertions considered to be moderately or severely painful, pain relief was provided 6.5% of the time, largely antispasmodics (3.5%) and NSAIDs (2.8%). Apart from pain relief, less than 1% of data were missing for all covariates (Table S1). More details on insertion characteristics by pain levels can be found in Table S2.

**TABLE 2:** Predictors of pain during IUD insertion (*n* = 7259), Campinas, Brazil.

Variables	All	Moderate or severe pain (*n* = 5910)	No moderate or severe pain (*n* = 1349)	Missing data	*p*‐value (χ ^2^)
	*n* (%)	*n* (%)	*n* (%)	(%)	
Age				0.7	**0.007**
18–24	2258 (31.3)	1882 (32.1)	376 (28.2)		
25–29	1628 (22.6)	1334 (22.7)	294 (22.1)		
30–34	1213 (16.8)	953 (16.2)	260 (19.5)		
35–39	1030 (14.3)	843 (14.4)	187 (14.0)		
40–45	1076 (14.9)	860 (14.7)	216 (16.2)		
Parity				0.5	**<0.001**
0	2648 (36.7)	2327 (39.5)	321 (24.1)		
1	1278 (17.7)	980 (16.6)	298 (22.4)		
2	1737 (24.1)	1367 (23.2)	370 (27.8)		
3+	1557 (21.6)	1214 (20.6)	343 (25.8)		
Any vaginal births				0.5	**<0.001**
Yes	3957 (54.8)	3116 (52.9)	841 (63.1)		
Any cesarean deliveries				0.5	**0.044**
Yes	2731 (37.8)	2195 (37.3)	536 (40.2)		
Insertion reason^a^					
Contraception	6287 (86.6)	5103 (86.4)	1184 (87.8)		0.166
AUB	978 (13.5)	746 (12.6)	232 (17.2)		**<0.001**
Endometriosis	189 (2.6)	153 (2.6)	36 (2.7)		0.868
Myoma	173 (2.4)	137 (2.3)	36 (2.7)		0.446
Other^b^	33 (0.5)	27 (0.5)	6 (0.4)		0.953
IUD type					0.695
Copper	911 (12.6)	746 (12.6)	165 (12.2)		
Hormonal	6348 (87.5)	5164 (87.4)	1184 (87.8)		
Pre‐insertion pain relief				3.4	**0.016**
Yes	429 (6.1)	367 (6.5)	62 (4.7)		
Type of pain relief^a^					
Analgesics	77 (1.1)	64 (1.1)	13 (1.0)		0.503
Antispasmodics	229 (3.2)	204 (3.5)	25 (1.9)		**0.026**
NSAIDs	204 (2.8)	168 (2.8)	36 (2.7)		0.073
Opioids	1 (0.0)	1 (0.0)	0		0.681
Healthcare Provider				0.2	0.134
Medical doctor	1955 (27.0)	1625 (27.5)	330 (24.5)		
OBGYN resident	2975 (41.1)	2395 (40.6)	580 (43.1)		
Medical student	116 (1.6)	93 (1.6)	23 (1.7)		
Nurse	2201 (30.4)	1787 (30.3)	414 (30.7)		
Insertion difficulty				0.8	**<0.001**
Any difficulty	506 (7.0)	378 (6.5)	128 (9.6)		
Use of cervical dilator				0.2	0.202
Yes	31 (0.4)	28 (0.5)	3 (0.2)		

*Note*: Significance level *p* ≤ 0.05.

Abbreviations: AUB, abnormal uterine bleeding; IUD, intrauterine device; OBGYN, obstetrician‐gynecologist.

^a^ Insertion reason and type of pain relief were multi‐select questions that enabled more than one answer per user, and each category was coded as binary (yes/no).

^b^ Other insertion reasons include post‐abortion, hematological disease, post transplanted solid organ, pre‐menstrual syndrome and hormone therapy after menopause.

Table 2 shows that individuals who experienced moderate to severe pain were on average younger, lower parity, and less likely to have previously had a vaginal or cesarean birth or to receive the IUD for abnormal uterine bleeding. These individuals were more likely to receive pre‐insertion pain relief, particularly antispasmodics, and providers were less likely to report difficulty during insertion.

Table 3 shows the unadjusted and adjusted association between each type of pain relief and moderate to severe pain. In the unadjusted model, antispasmodics were associated with higher odds of moderate to severe pain (OR = 1.87; 95% confidence interval [CI]: 1.23–2.84). We further adjusted for age and parity, as adjusting for cesarean deliveries, insertion difficulty, and insertion provider did not affect our odds ratio. After adjusting for age and parity, we found strong evidence that pre‐insertion antispasmodics were associated with moderate to severe pain (adjusted OR = 1.75; 95% CI: 1.15–2.68). We found no evidence that NSAIDs were associated with moderate or severe pain.

**TABLE 3:** Type of pre‐insertion pain relief distribution, by pain level (*n* = 6931), Campinas, Brazil.

Pre‐insertion medications	Moderate or severe pain	Crude OR (95% CI)	Adjusted OR (95% CI)^a^
	*n*/*N* (%)		
Type of pain relief^b^		*P* = 0.849	*P* = 0.975
Any analgesics	60/73 (82.2)	1.06 (0.58–1.94)	0.99 (0.54–1.82)
No analgesics	5577/6858 (81.3)	1	1
		*P* = 0.004	*P* = 0.009
Any antispasmodics	200/225 (88.9)	1.87 (1.23–2.84)	1.75 (1.15–2.68)
No antispasmodics	5437/6706 (81.1)	1	1
		*P* = 0.643	*P* = 0.694
Any NSAIDs	166/201 (82.6)	1.09 (0.75–1.58)	0.93 (0.64–1.35)
No NSAIDs	5471/6730 (81.3)	1	1

Abbreviations: CI, confidence interval; NSAIDS, non‐steroidal anti‐inflammatory drugs; OR, odds ratio.

^a^ Adjusted for age (a priori) and parity.

^b^ Pre‐insertion pain relief type was defined as any insertion in which the specified medication class was used, either alone or in combination with other medications, versus insertions without that medication class, including those using other pain relief medications or none.

## 4 DISCUSSION

In this large, real‐world analysis from a Brazilian public teaching hospital, we found that moderate to severe pain was experienced in over 80% of IUD insertions. Around 6% of insertions were done with oral pain relief (largely antispasmodics and NSAIDs). We found evidence that pre‐insertion antispasmodics were associated with higher odds of moderate to severe pain.

Moderate to severe pain accounted for 81% of all IUD insertions. This is higher than other Brazilian studies in different centers that followed similar guidelines and assessment methods, where moderate to severe pain varied from 44% to 70% of insertions.^3^, ^10^, ^11^, ^16^ Although differences in inserter design and insertion technique might contribute to pain perception, causal inferences cannot be drawn from this observational analysis. The center inserts a high proportion of hormonal IUDs, with a larger inserter tube than those used in the private sector, which might help explain the higher pain scores observed.^4^, ^5^ Smaller sample sizes and higher proportions of parous individuals in other studies might also explain the differences in pain.^10^, ^11^

Our analysis found higher odds of moderate to severe pain when antispasmodics were administered. Antispasmodics are commonly used before IUD insertions, yet evidence on their effectiveness in reducing discomfort is limited, with studies reporting no benefit with increased post‐insertion pain.^17^, ^18^ Our findings might have been influenced by residual confounding, where antispasmodics might have been used in more complex cases, and/or by individuals with higher levels of anticipated pain, which might be driving this association.^5^ Our results on other pain relief medications are aligned with previous findings that suggest oral analgesia is not associated with decreased insertion‐related pain.^15^, ^17^, ^19^ Finally, it is important to clarify that the center does not routinely employ topical lidocaine formulations and paracervical block, interventions that may effectively decrease insertion pain.^5^, ^20^, ^21^

The clinic already provides sexual and reproductive health guidance, offering users resources to make informed decisions about their preferred method. To improve patient experience, we recommend standardizing protocols to address realistic pain expectations, acknowledge concerns about discomfort, and offer patient‐centered options for pain management.^5^, ^6^

Our study was performed in a public facility. Contraceptive care differs greatly in private facilities in Brazil, where multiple IUD types and pain relief options are available on demand.^22^ White women, those with more years of schooling, and higher socioeconomic status know more about IUDs and have greater access to private care.^22^, ^23^ In contrast, Black, Brown and Indigenous women have experienced more contraceptive coercion, have poorer access to long‐acting reversible contraceptives, and have their pain underestimated and undertreated more frequently.^22^, ^24^ Our analysis lacked data on race/ethnicity and sociodemographic status, and future research should assess differences in pain and pain management based on these characteristics.

Future research should adjust for anxiety and anticipated pain as potential confounders. Differences in practices between private and public settings should also be examined, including contraceptive counseling, method choice, and pain relief options in Brazil. Finally, non‐invasive pain management interventions should be further explored to improve existing evidence and prevent the use of painful injectable techniques.

Our study's strengths include a large sample size of individual‐level data collected from health records reflecting routine healthcare practices. Our analysis had few missing values, which helped minimize bias, and provided an accurate representation of the population of interest. We employed a widely used self‐report tool for insertion pain, enabling comparability with similar studies. Additionally, recall bias was reduced by immediate collection of pain scores after insertion.

Our study had some weaknesses. Our study included data from a single center in Campinas, a municipality that has a very high Municipal Human Development Index; with great disparities in income, schooling, and longevity when compared to other areas in Brazil.^25^ Additionally, no other sociodemographic variables, such as race/ethnicity and socioeconomic status were assessed. We lacked the power to examine individual medications, which could have varying associations with pain due to differing mechanisms of action, half‐life, and excretion. The exact timing of pain relief administration was not recorded, yet the interval between intake and the start of the procedure might affect pain management coverage. Moreover, as the same providers who performed insertion also collected the pain scores, pain might have been underreported as we cannot exclude courtesy bias. Finally, this study did not measure anticipated pain and anxiety, comorbidities, breastfeeding status, insertion timing or interval since last birth, variables that have been found to influence pain outcomes.^5^

Even though public attention to IUD insertion has increased recently, this matter has been a subject of scientific investigation for over 40 years.^19^ However, it is still not properly addressed by available interventions and clinical guidance in contraceptive care. While Brazilian guidelines^9^ suggest that up to 5% of individuals experience moderate to severe pain, our findings show that these levels were experienced in over 80% of IUD insertions. Therefore, we recommend that national guidelines are updated to reflect realistic pain experiences, inform pain management practices, and promote patient‐centered counseling in contraceptive care. In conclusion, we argue that improving pain management and user experiences during IUD insertions is imperative, as it could increase IUD acceptance, reduce barriers to uptake, and encourage more individuals to consider its use.

## AUTHOR CONTRIBUTIONS

ALS, JL, CRTJ, RS, and LB provided substantial contributions to the conception of the work, the acquisition of the data, analysis, and interpretation of data for the work; participated in drafting the work and revising it critically for important intellectual content; and provided final approval of the version to be published. Authors agree to be accountable for all aspects of the work and ensure that questions related to the accuracy or integrity of any part of the work are appropriately investigated and resolved.

## FUNDING INFORMATION

Our study received partial financial support from the *Fundação de Amparo à Pesquisa do Estado de São Paulo* (FAPESP) award No. 2023/13279–5. The study funder had no role in the study design, data collection, analysis, interpretation, report writing, or decision to submit the manuscript. This article represents the views of the named authors only and does not represent the views of the mentioned organizations. The hormonal devices were donated by the International Contraceptive Access Foundation, Turku, Finland, under an unrestricted grant. The copper devices were donated by Injeflex, São Paulo, Brazil.

## CONFLICT OF INTEREST STATEMENT

LB served on Medical Advisory Boards and has given ad hoc lectures for Organon and Bayer. CRTJ previously received an honorarium from Organon and Bayer as speaker. The other authors declare no conflict of interest.

## Supporting information


**Figure S1.** Directed acyclic graph of the association between pre‐insertion pain relief and moderate to severe pain during IUD insertion.
**Table S1.** Covariates with missing data.
**Table S2.** Insertion characteristics, by pain levels (n = 7259).
**Table S3.** Pain relief medication (n = 7259).

## Data Availability

The datasets used and/or analyzed during the current study are registered at the *Repositório de Dados de Pesquisa da UNICAMP* (*REDU*) under the following DOI: https://doi.org/10.25824/redu/RKKPKW.
