# Supplementary material for: Pain at intrauterine device placement in a Brazilian center
Source: Int J Gynaecol Obstet. 2026 Apr 21;175(1):358–64. doi: 10.1002/ijgo.71026 (PMC13629569; doi:10.1002/ijgo.71026)
Supplement: Supplementary file 1 [file IJGO-175-358-s001.docx]

# SUPPLEMENTARY FILES

**FIGURES**

**Figure S1: Directed acyclic graph of the association between pre-insertion pain relief and moderate to severe pain during IUD insertion**

**TABLES**

**Table S1: Covariates with missing data**

| *Variable* | Missing Values | | |
| --- | --- | --- | --- |
|  | All  (n =7,259) | Moderate or severe pain  (n=5,910) | No moderate or severe pain  (n=1,349) |
|  | *n (%)* | *n (%)* | *n (%)* |
| Age | 54 (0.7) | 38 (0.6) | 16 (1.2) |
| Parity | 39 (0.5) | 22 (0.4) | 17 (1.3) |
| Any vaginal births | 39 (0.5) | 22 (0.4) | 17 (1.3) |
| Any cesarean deliveries | 39 (0.5) | 22 (0.4) | 17 (1.3) |
| Insertion reason^a^ | 0 (0) | 0 (0) | 0 (0) |
| IUD Type | 0 (0) | 0 (0) | 0 (0) |
| Pre-insertion pain relief | 243 (3.4) | 217 (3.7) | 26 (1.9) |
| Provider | 12 (0.2) | 10 (0.2) | 2 (0.2) |
| Insertion difficulty | 60 (0.8) | 46 (0.8) | 14 (1.0) |
| Use of cervical dilator | 14 (0.2) | 11 (0.2) | 3 (0.2) |

^a^ Insertion reason was a multi-select question that enabled more than one answer per individual. Each variable for insertion reason was coded as yes or no, and did not account for missing data.

**Table S2: Insertion characteristics, by pain levels (n=7,259)**

| *Characteristic* | All | No pain  (n=228) | Mild pain  (n=1,121) | Moderate pain  (n=2,003) | Severe pain  (n=3,907) |
| --- | --- | --- | --- | --- | --- |
|  | *n (%)* | *n (%)* | *n (%)* | *n (%)* | *n (%)* |
| *Age* |  |  |  |  |  |
| 18-24 | 2,258 (31.3) | 57 (25.6) | 319 (28.7) | 574 (28.9) | 1,308 (33.7) |
| 25-29 | 1,628 (22.6) | 46 (20.6) | 248 (22.3) | 467 (23.5) | 867 (22.3) |
| 30-34 | 1,213 (16.8) | 42 (18.8) | 218 (19.6) | 354 (17.8) | 599 (15.4) |
| 35-39 | 1,030 (14.3) | 40 (17.9) | 147 (13.2) | 298 (15.0) | 545 (14.0) |
| 40-45 | 1,076 (14.9) | 38 (17.0) | 178 (16.0) | 294 (14.8) | 566 (14.6) |
| *Parity* |  |  |  |  |  |
| 0 | 2,648 (36.7) | 40 (18.1) | 281 (25.3) | 599 (30.0) | 1,728 (44.4) |
| 1 | 1,278 (17.7) | 46 (20.8) | 252 (22.7) | 378 (19.0) | 602 (15.5) |
| 2 | 1,737 (24.1) | 62 (28.1) | 308 (27.7) | 516 (25.9) | 851 (21.9) |
| 3+ | 1,557 (21.6) | 73 (33.0) | 270 (24.3) | 501 (25.1) | 713 (18.3) |
| *Any vaginal births* |  |  |  |  |  |
| Yes | 3,957 (54.8) | 159 (72.0) | 682 (61.4) | 1,239 (62.1) | 1,877 (48.2) |
| *Any cesarean deliveries* |  |  |  |  |  |
| Yes | 2,731 (37.8) | 93 (42.1) | 443 (39.9) | 814 (40.8) | 1,381 (35.5) |
| *Insertion reason* ^a^ |  |  |  |  |  |
| Contraception | 6,287 (86.6) | 199 (87.3) | 985 (87.9) | 1,757 (87.7) | 3,346 (85.6) |
| AUB | 978 (13.5) | 29 (12.7) | 203 (18.1) | 239 (11.9) | 507 (13.0) |
| Endometriosis | 189 (2.6) | 5 (2.2) | 31 (2.8) | 44 (2.2) | 109 (2.8) |
| Myoma | 173 (2.4) | 4 (1.8) | 32 (2.9) | 43 (2.2) | 94 (2.4) |
| Other ^b^ | 33 (0.5) | 0 (0.0) | 6 (0.5) | 11 (0.6) | 16 (0.4) |
| *IUD Type* |  |  |  |  |  |
| Copper | 911 (12.6) | 44 (19.3) | 121 (10.8) | 303 (15.1) | 443 (11.3) |
| Hormonal | 6,348 (87.5) | 184 (80.7) | 1,000 (89.2) | 1,700 (84.9) | 3,464 (88.7) |
| *Pre-insertion pain relief* |  |  |  |  |  |
| Yes | 429 (6.1) | 8 (3.6) | 54 (4.9) | 107 (5.5) | 260 (7.0) |
| *Pain Relief Combination* ^c^ |  |  |  |  |  |
| 1 Type | 347 (80.9) | 7 (87.5) | 43 (79.6) | 86 (80.4) | 211 (81.2) |
| 2 Types | 82 (19.1) | 1 (12.5) | 11 (20.4) | 21 (19.6) | 49 (18.9) |
| *Provider* |  |  |  |  |  |
| Medical doctor | 1,955 (27.0) | 59 (25.9) | 271 (24.2) | 546 (27.3) | 1,079 (27.7) |
| OBGYN resident | 2,975 (41.1) | 103 (45.2) | 477 (42.6) | 922 (46.2) | 1,473 (37.7) |
| Medical student | 116 (1.6) | 5 (2.2) | 18 (1.6) | 44 (2.2) | 49 (1.3) |
| Nurse | 2,201 (30.4) | 61 (26.8) | 353 (31.6) | 485 (24.3) | 1,302 (33.4) |
| *Insertion difficulty* |  |  |  |  |  |
| Any difficulty | 506 (7.0) | 3 (1.3) | 125 (11.3) | 86 (4.3) | 292 (7.5) |
| *Use of cervical dilator* |  |  |  |  |  |
| Yes | 31 (0.4) | 2 (0.9) | 1 (0.1) | 6 (0.3) | 22 (0.6) |

^a^ Insertion reason and type of pain relief were multi-select questions that enabled more than one answer per user, and each category was coded as binary (yes/no). ^b^ Other insertion reasons include post-abortion, hematological disease, post transplanted solid organ, pre-menstrual syndrome and hormone therapy after menopause. ^c^ Percentages are calculated based on those who received any pain relief.

**Table S3: Pain relief medication (n=7,259)**

| *Medication classes* | Medication Name (Portuguese) | Active Ingredients/Category |
| --- | --- | --- |
| Analgesics | Beserol | Caffeine + Carisoprodol + Diclofenac + Acetaminophen |
|  | Dipirona | Metamizole |
|  | Dorflex | Metamizole + Orphenadrine citrate + Caffeine |
|  | Lisador | Metamizole + Adiphenine hydrochloride + Promethazine hydrochloride |
|  | Paracetamol | Paracetamol |
| Antispasmodics | Buscopan | Scopolamine butylbromide |
|  | Buscopan/Neocopan Composto | Metamizole + Scopolamine butylbromide |
|  | Butilbrometo de Escopolamina | Scopolamine butylbromide |
|  | Hioscina | Scopolamine butylbromide |
|  | Tropinal | Metamizole + Scopolamine butylbromide + Hyoscine butylbromide + Homatropine methylbromide |
| Non-Steroidal Anti-Inflammatory Drugs (NSAIDs) | Aceclofenaco | Aceclofenac |
|  | Ácido Mefenâmico | Mefenamic Acid |
|  | Advil | Ibuprofen |
|  | Anti-inflamatórios Não Esteróides | NSAIDs |
|  | Beserol | Caffeine + Carisoprodol + Diclofenac + Acetaminophen |
|  | Buscofem | Ibuprofen |
|  | Celecoxibe | Celecoxib |
|  | Cetoprofeno | Ketoprofen |
|  | Diclofenaco | Diclofenac |
|  | Espidufen | Ibuprofen |
|  | Ibuprofeno | Ibuprofen |
|  | Naproxeno | Naproxen |
|  | Nimesulida/Nivux | Nimesulide |
|  | Ponstan | Mefenamic Acid |
|  | Proflam | Aceclofenac |
|  | Tandrilax | Caffeine + Carisoprodol + Diclofenac + Paracetamol |
|  | Teflan | Tenoxicam |
|  | Tenoxicam | Tenoxicam |
|  | Toragesic | Ketorolac tromethamine |
|  | Tormiv | Ketorolac tromethamine |
|  | Trometamol Cetorolaco | Ketorolac tromethamine |
|  | Vóric | Ketorolac tromethamine |
| Opioids | Tramadol | Tramadol |
|  | Tramal | Tramadol |
